# Acute Kidney Injury in Patients With Cirrhosis Exposed to Iodinated Contrast: A Systematic Review and Meta‐Analysis

**DOI:** 10.1002/jgh3.70474

**Published:** 2026-09-18

**Authors:** Tulio L. Correa, Vanio Antunes, Otavio C. Martins, Matheus V. Fernandes, Elisio Bulhoes, Alonzo A. Prata, Ana Carolina Covre Coan, André Milani Reis, Natalia J. Milioli, Ximena Parraga, Beatriz Sordi Chara, Cristiane Valle Tovo

**Affiliations:** ^1^ Division of General Internal Medicine University of Pittsburgh Medical Center Pittsburgh Pennsylvania USA; ^2^ Federal University of Health Sciences of Porto Alegre Porto Alegre Brazil; ^3^ Division of Gastroenterology and Hepatology Mayo Clinic Rochester Minnesota USA; ^4^ College of Higher Education of the United Amazon Redenção Brazil; ^5^ Federal University of Espirito Santo Vitoria Brazil; ^6^ State University of Campinas Campinas Brazil; ^7^ Pontifical Catholic University of Rio Grande do Sul Porto Alegre Brazil; ^8^ Division of Gastroenterology, Hepatology, and Nutrition Virginia Commonwealth University Medical Center Richmond Virginia USA; ^9^ Yale New Haven Hospital, Yale School of Medicine New Haven Connecticut USA

## Abstract

**Introduction:**

Iodinated radiocontrast agents are widely used in diagnostic radiography and therapeutic procedures. However, concerns remain regarding their association with contrast‐induced acute kidney injury (CI‐AKI) and contrast‐associated acute kidney injury (CA‐AKI). While these risks are frequently discussed in the general population, data on CI and CA‐AKI in patients with cirrhosis remains limited.

**Aims:**

Evaluate the incidence and risk factors associated with CI‐AKI or CA‐AKI in patients with cirrhosis exposed to iodinated contrast (IC).

**Methods:**

A systematic search was conducted in PubMed, Embase, and Cochrane Central for studies including patients with cirrhosis exposed to IC. Binary endpoints were assessed using pooled odds ratios (OR), and continuous endpoints using mean differences (MDs) or standardized mean differences (SMDs). The primary outcome was the pooled incidence of AKI in patients with cirrhosis exposed to IC, with secondary outcomes including the risk of CI‐AKI compared to unexposed patients and risk factors associated with CA‐AKI development. Analyses were performed in R (version 4.3.2).

**Results:**

Nine observational studies were included, two of which used propensity score matching, encompassing a total of 2389 patients with cirrhosis, of whom 1672 were exposed to IC. The pooled incidence of acute kidney injury was 11.1% (95% CI 5.3%–18.8%). On the leave‐one‐out analysis, patients with cirrhosis receiving IC had a higher risk of AKI compared to those not receiving IC (OR 2.5; 95% CI 1.4–4.4). The presence of ascites was significantly associated with the development of CA‐AKI (OR 3.2; 95% CI 1.8–5.5). Those who developed CA‐AKI had significantly higher baseline MELD scores, associated with higher serum bilirubin and INR levels.

**Conclusion:**

Patients with cirrhosis exposed to IC agents had a higher risk of developing acute kidney injury compared to those not exposed. The development of CA‐AKI was significantly associated with the presence of ascites and elevated baseline MELD scores, along with higher serum bilirubin and INR levels.

**Abbreviations**
AKI: acute kidney injury
CA‐AKI: contrast‐associated acute kidney injury
CI‐AKI: contrast‐induced acute kidney injury
eGFR: estimated glomerular filtration rate
ESLD: end‐stage liver disease
HCC: hepatocellular carcinoma
IC: iodinated contrast
INR: internalized normalized ratio
MELD: model for end‐stage liver disease
PRISMA: Preferred Reporting Items for Systematic reviews and Meta‐Analyses

## 1 Introduction

Iodinated radiocontrast agents are widely used in diagnostic radiography and therapeutic procedures [1]. However, concerns have long been raised about their potential nephrotoxic effects [2]. Contrast‐induced acute kidney injury (CI‐AKI) refers to a decline in kidney function following the recent intravascular administration of contrast agents for imaging or interventional purposes, after excluding other potential causes of acute kidney injury (AKI) [3]. Meanwhile, contrast‐associated acute kidney injury (CA‐AKI) is a broader term that encompasses any AKI event occurring within 48 h after contrast media administration [4].

Recent data suggest that the incidence of CI‐AKI may be lower than initially presumed [5]. A meta‐analysis of 25 950 patients showed a similar incidence of AKI, need for kidney replacement therapy, and mortality between the medium‐dose contrast group and the control group [6]. However, studies predominantly include patients without liver disease, and evidence on the incidence of CI‐AKI in patients with cirrhosis remains limited.

AKI occurs in up to 60% of hospitalized patients with cirrhosis [7]. Patients with cirrhosis who develop AKI have increased risks of short‐term and long‐term mortality, with the prognosis depending on the severity of both kidney and liver dysfunction [7]. When contrast agents are administered, patients with advanced liver disease may be particularly vulnerable to contrast‐associated kidney damage, resulting in higher rates of CI‐AKI [8]. In this study, we conducted a systematic review and meta‐analysis to assess the outcomes and identify risk factors associated with CI and CA‐AKI in patients with cirrhosis exposed to iodinated contrast (IC).

## 2 Methods

This systematic review with meta‐analysis was registered in the international prospective register of systematic reviews (PROSPERO) under the protocol number CRD420251011563. This study was designed and conducted according to the Preferred Reporting Items for Systematic Reviews and Meta‐analyses (PRISMA) reporting guidelines [9].

### 2.1 Study Eligibility

Inclusion in this meta‐analysis was restricted to studies that met all the following eligibility criteria: (1) randomized or observational studies; (2) studies providing data on patients with cirrhosis receiving IC; (3) full‐text availability; and (4) studies reporting at least one of the clinical outcomes of interest. We excluded (1) studies that did not report AKI according to a prespecified diagnostic definition or provide sufficient data to calculate AKI incidence; (2) studies in which renal function was not assessed within an appropriate timeframe following contrast administration; (3) studies not designed to analyze cirrhotic patients as a distinct group; and (4) conference abstracts, letters to the editor, reviews, and meta‐analyses. In cases of overlapping study populations, only the study with the largest patient cohort was included.

Two authors (T.L.C. and E.B.) independently screened by title and abstract following predefined search criteria and full text review. Disagreements were resolved by consensus or, if necessary, by a third evaluator (V.A.).

### 2.2 Search Strategy and Data Extraction

We conducted a systematic search of PubMed, Embase, and the Cochrane Library in June 2026 using the following search terms: “cirrhosis,” “AKI,” “acute kidney injury,” and “iodinated contrast”. In addition, the references of the included manuscripts were evaluated to identify additional relevant studies. Data were extracted on study characteristics: (1) name, (2) year, (3) country, (4) design, and (5) sample size. Patient baseline characteristics collected included (1) mean age, (2) gender distribution, (3) mean MELD score, (4) eGFR, and (5) volume of applied IC (Table 1).

**TABLE 1:** Baseline characteristics of the included studies.

Study	Design	Country	Cirrhosis study population	Groups	Sample size	Male (%)	Age (years)	Meld score	Child–Pugh score	eGFR (MDRD‐4)	Dose of IC	HCC (%)
Abideen, 2018	Retrospective	Pakistan	Patients with liver cirrhosis who had a CECT	CI‐AKI No CI‐AKI	24 446	66 84	48.67 ± 4.59 49.41 ± 9.78	21.67 ± 6.47 17.04 ± 5.52	10.00 ± 1.67 9.26 ± 1.80	82.29 ± 19.03 95.11 ± 27.93	103.00 ± 15.44 mL 106.46 ± 21.67 mL	0 32
Bhandari, 2019	Retrospective	United States	ESLD patients exposed to cardiac catheterizations	Non‐CI‐AKI	138	69.6	60.3 ± 9.0	9.6 ± 5.7	—	70.3 ± 24.9	114.0 ± 86.5 mL	—
				CI‐AKI	41	73.2	59.1 ± 10.1	11.6 ± 7.7	—	77.8 ± 31.4	119.6 ± 125.4 mL	—
Campion, 2023	PSM	Italy	Patients with liver cirrhosis undergoing CECT	Cirrhosis + CECT	148	74	61 (54–67)^a^	14 (11–18)^a^	A: 42 (28.4%) B: 70 (47.3%) C: 36 (24.3%)	97.7 (75.0–125.9)^a^	50 (44–56) g^a^	—
				Cirrhosis, no CECT	133	60	59 (52–68)^a^	16 (13–20)^a^	A: 11 (8.3%) B: 70 (52.6%) C: 52 (39.1%)	86.4 (61.0–113.6)^a^	0	—
				No cirrhosis + CECT	163	58	66 (54–78)^a^	—	—	94.0 (75.9–118.6)^a^	44 (41–48) g^a^	—
Filomia, 2016	Retrospective	Italy	Patients with liver cirrhosis undergoing CECT	CECT group	249	66.3	64 ± 11	9 ± 3	A: 143 (57.4%) B: 88 (35.3%) C: 18 (7.2%) A: 96 (47.3%)	83 ± 30	—	34.5
				No CECT	203	65	66 ± 10	11 ± 4	B: 84 (41.4%)	72 ± 29	0	26.1
									C: 23 (11.3%)			
Khan, 2020	Retrospective	United States	Patients with cirrhosis who underwent multiphase multidetector CECT or MRI	CECT MRI	173 243	64.1 66.2	62.26 ± 11.88 62.81 ± 10.19	13.92 ± 6.62 13.57 ± 6.70	A: 170 (40.89%) B: 137 (32.89%) C: 109 (26.22%)	— —	— 0	— —
Najjar, 2002	Case–Control	United States	Patients with and without cirrhosis exposed to IC	Cirrhosis	72	38 (58%)	55 ± 1.6	—	—	—	—	—
				No cirrhosis	72	40 (61%)	48 ± 2.4	—	—	—	—	—
Lodhia, 2009	Retrospective	United States	Patients with cirrhosis undergoing CECT	CIN	53	—	51.9	17	—	—	—	—
				No‐CIN	163	—	53.6	14.8	—	—	—	—
Safi, 2015	Retrospective	Germany	Patients with cirrhosis undergoing imaging evaluation	IC	84	57 (67.9)	61.7 ± 11.2	13.8 ± 5.1	—	—	—	24 (28.9)
				No IC	68	49 (72.1)	56.9 ± 9.6	13.7 ± 6.1	—	—	—	21 (30.9)
Tergast, 2022	PSM	Germany	Decompensated liver cirrhosis patients admitted to the hospital	CM‐CT	51	73	61 ± 11	18 ± 6	—	26 ± 7	—	—
				No CM‐CT	100	71	57 ± 11	22 ± 6	—	25 ± 10	0	—

*Note:* Continuous data are shown as mean and standard deviation unless otherwise specified.

Abbreviations: CECT = contrast‐enhanced computed tomography; CI‐AKI = contrast‐induced acute kidney injury; CIN = contrast‐induced nephropathy; CM‐CT = contrast‐medium computed tomography; CP = Child–Pugh; ESLD = end‐stage liver disease; HCC = hepatocellular carcinoma; IC = iodinated contrast; LC = liver cirrhosis; MRI = magnetic resonance imaging; PSM = propensity‐score matching.

^a^ Median (IQR).

Two authors (V.A. and M.V.) independently extracted the data following predefined search criteria and quality assessment. Disagreements were resolved by consensus or, if necessary, by a third evaluator (T.C.).

### 2.3 Endpoints

In line with the Consensus Statements from the American College of Radiology and the National Kidney Foundation [4], we classified studies involving a well‐matched control group without exposure to IC as CI‐AKI. In contrast, studies lacking a control group are categorized as CA‐AKI due to insufficient evidence to establish a causal relationship.

We conducted four main analyses. First, we performed a single‐arm meta‐analysis to evaluate the development of AKI in all patients with cirrhosis exposed to IC. In the second analysis, we carried out a pairwise meta‐analysis to assess the impact of IC exposure in the development of CI‐AKI in patients with cirrhosis. Third, we conducted a pairwise meta‐analysis to evaluate the impact of IC exposure on the development of AKI in patients with cirrhosis analysis to compare baseline characteristics; including the presence of ascites, diuretic use, chronic kidney disease (CKD), male sex, diabetes mellitus, mean model for end‐stage liver disease (MELD) score, serum bilirubin levels, and international normalized ratio (INR) between patients with cirrhosis who developed AKI and those who did not. Lastly, we performed a pairwise meta‐analysis to evaluate cirrhosis as a risk factor for the development of CI‐AKI in patients exposed to IC.

### 2.4 Quality Assessment

The methodological quality of all included studies was evaluated independently by two authors (O.M. and A.C.), following the Cochrane Collaboration tool. We assessed the risk of bias in all studies using the ROBINS‐I for non‐randomized studies [10]. The risk of bias plots was created with the Robvis tool. Disagreements were resolved by consensus or, if necessary, evaluated by a third author (V.A.). Funnel plots and Egger's test were not constructed to examine the publication bias of the single‐arm meta‐analysis on AKI incidence due to insufficient number of studies.

### 2.5 Sensitivity Analysis

A subgroup analysis was conducted on the overall incidence of AKI, stratified by the definitions of CA‐AKI and CI‐AKI, and the presence of matched controls. Additionally, a leave‐one‐out analysis was performed for all outcomes with moderate or high heterogeneity to identify potential sources of variability and assess the influence of individual studies.

### 2.6 Data Analysis

For binary endpoints, treatment effects were assessed using pooled odds ratios (ORs) in pairwise comparisons, while arcsine transformation along with the inverse variance were employed for meta‐analysis of proportions. For continuous endpoints, the weighted mean difference (MD) was calculated. All treatments were evaluated with 95% confidence intervals (CIs). *p* < 0.05 were considered statistically significant. When available, univariate analysis of binary data was used to assess the treatment effect of risk factors for the development of AKI. Due to varying cut‐offs among studies, we chose not to pool ORs for continuous data.

Given the anticipated heterogeneity across observational studies, we applied a restricted maximum likelihood random‐effects model for the pooled analysis. Heterogeneity was assessed using *I* ^2^ statistics. All statistical analyses were conducted using the R program (version 4.3.2) with the *meta* package.

## 3 Results

### 3.1 Study Selection and Baseline Characteristics

**FIGURE 1:**
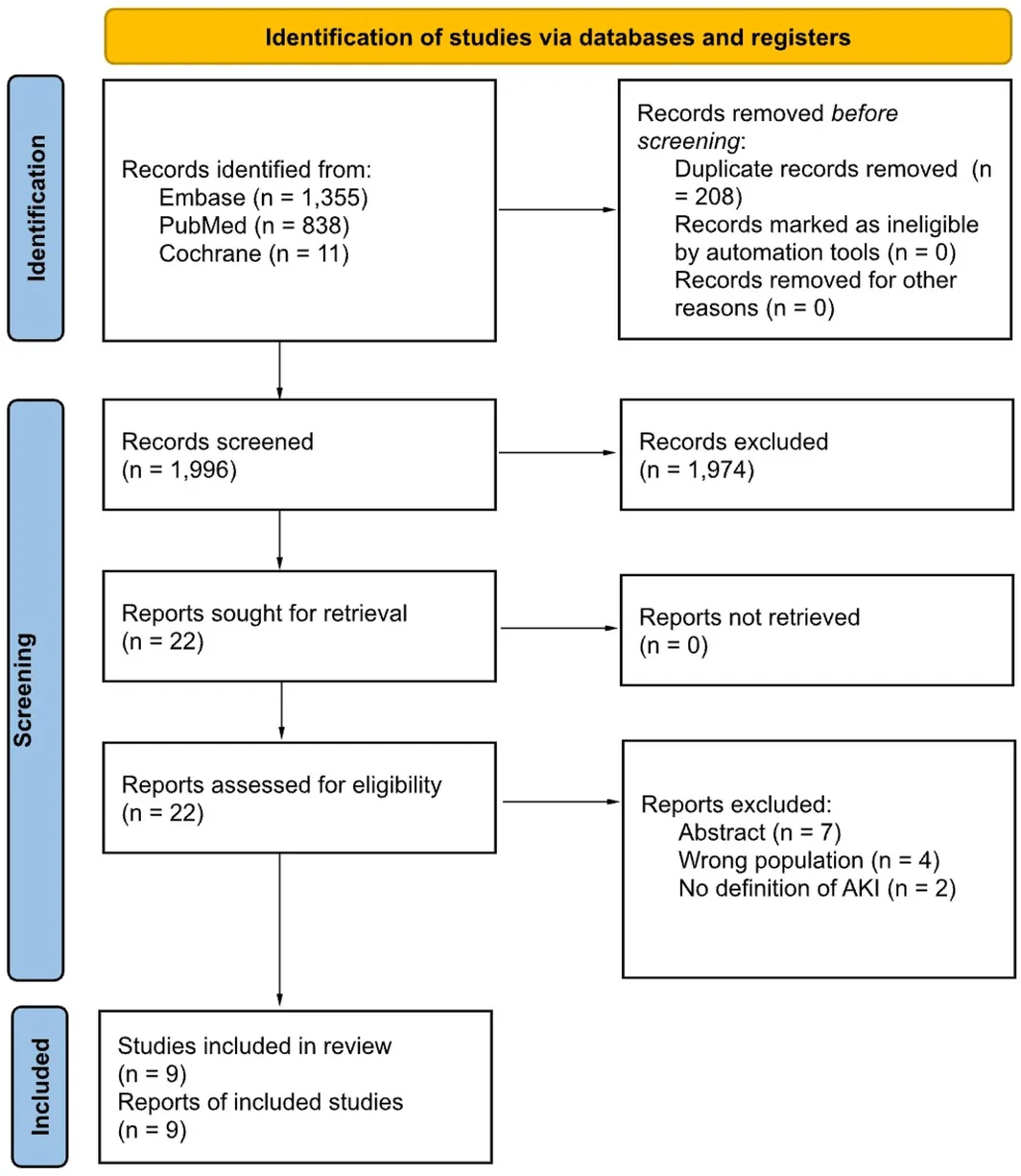
PRISMA flow diagram of study screening and selection.

A total of 2204 articles were initially identified through our search strategy, as detailed in Figure 1. After removing duplicates and studies based on title/abstract review, 22 articles remained for full‐text review. Ultimately, nine studies were included in this systematic review [8, 11, 12, 13, 14, 15, 16, 17, 18]. All included studies were observational in design, including two propensity score‐matched analyses. The study populations represented a broad spectrum of cirrhosis severity; although most studies did not stratify patients by compensated versus decompensated disease, available Child–Pugh data suggested a predominance of Child–Pugh B/C cirrhosis across most cohorts. Specific definitions of AKI also varied among the included studies due to changes in diagnostic criteria over time and are summarized in Table S1.

### 3.2 Prevalence of AKI in Patients With Cirrhosis Exposed to Iodinated Contrast

**FIGURE 2:**
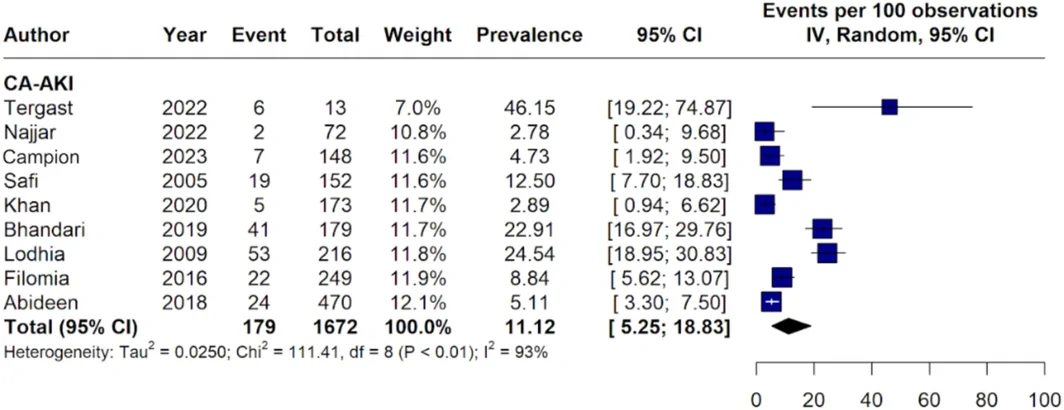
Prevalence of CA‐AKI in patients with cirrhosis exposed to iodinated contrast.

The prevalence of AKI, regardless of its definition, was reported in all nine studies, including 179 events in 1672 patients with cirrhosis exposed to IC. The analysis showed a pooled prevalence of 11.1% (95% CI 5.3%–18.8%; *I* ^2^ = 93%; Figure 2).

### 3.3 CI‐AKI Risk in Patients With Cirrhosis Following Iodinated Contrast Exposure

**FIGURE 3:**
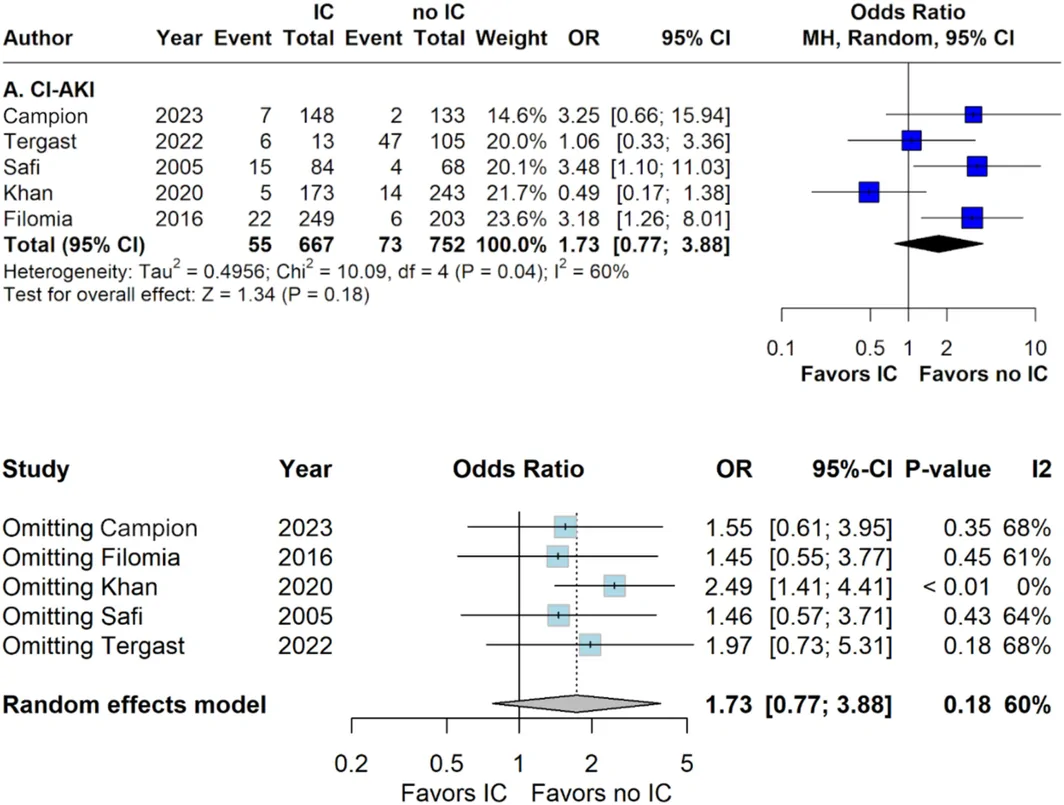
Impact of iodinated contrast exposure on CI‐AKI risk in patients with cirrhosis, including leave‐one‐out sensitivity analysis.

On initial analysis, patients with cirrhosis who received IC did not show a significantly higher risk of developing CI‐AKI compared to those who did not receive IC (OR 1.7; 95% CI 0.8–3.9; *I* ^2^ = 60%; Figure 3A). However, a leave‐one‐out analysis identified the study by Khan et al. as the main source of heterogeneity [14]. Excluding this study notably altered the results and suggested that patients who received IC had a significantly higher risk of AKI (OR 2.5; 95% CI 1.4–4.4; *I* ^2^ = 0%; Figure 3B).

### 3.4 Risk Factors for CA‐AKI in Patients With Cirrhosis Exposed to Iodinated Contrast

**FIGURE 4:**
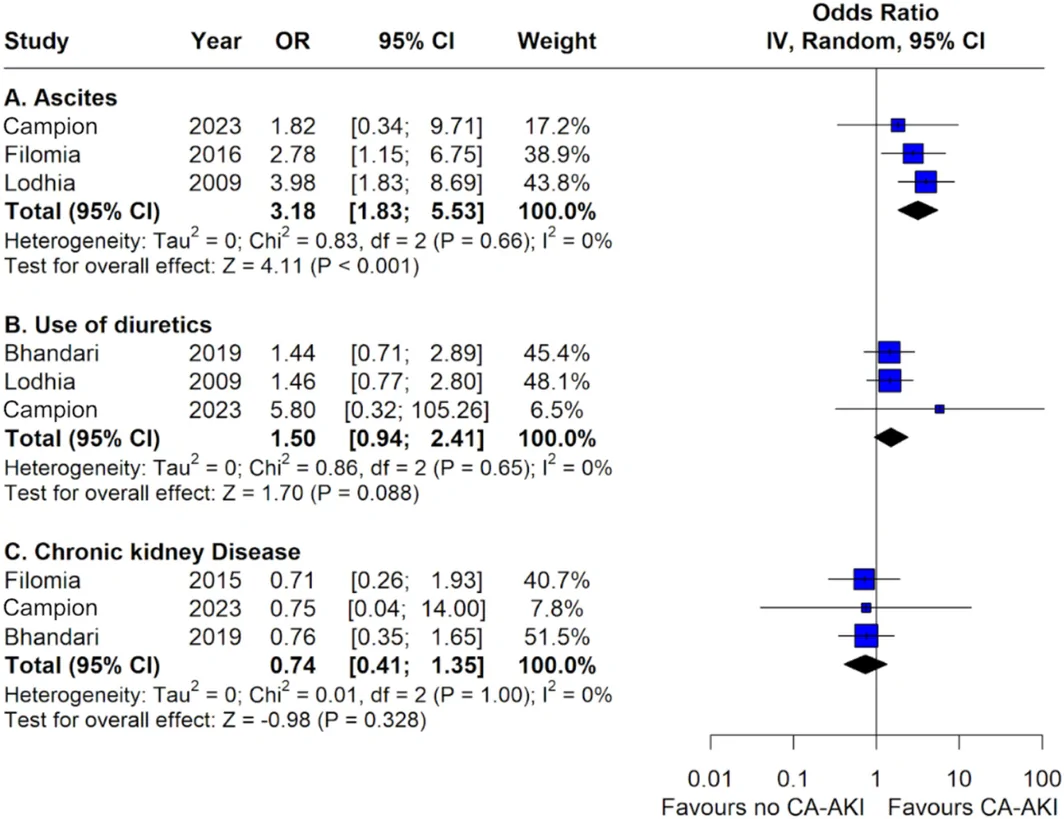
Comparison of baseline ascites, diuretic use, and chronic kidney disease between iodinated contrast‐exposed patients with cirrhosis who developed CA‐AKI versus those who did not.

In the pooled analysis, the presence of ascites was significantly associated with the development of CA‐AKI (OR 3.2; 95% CI 1.8–5.5; *I* ^2^ = 0%; Figure 4A) in the three studies that evaluated it. No significant association was found between the baseline use of diuretics (OR 1.5; 95% 0.9–2.4; *I* ^2^ = 0%; Figure 4B) or the baseline presence of CKD (OR 0.7; 95% 0.4–1.4; *I* ^2^ = 0%; Figure 4C) and the development of CA‐AKI.

**FIGURE 5:**
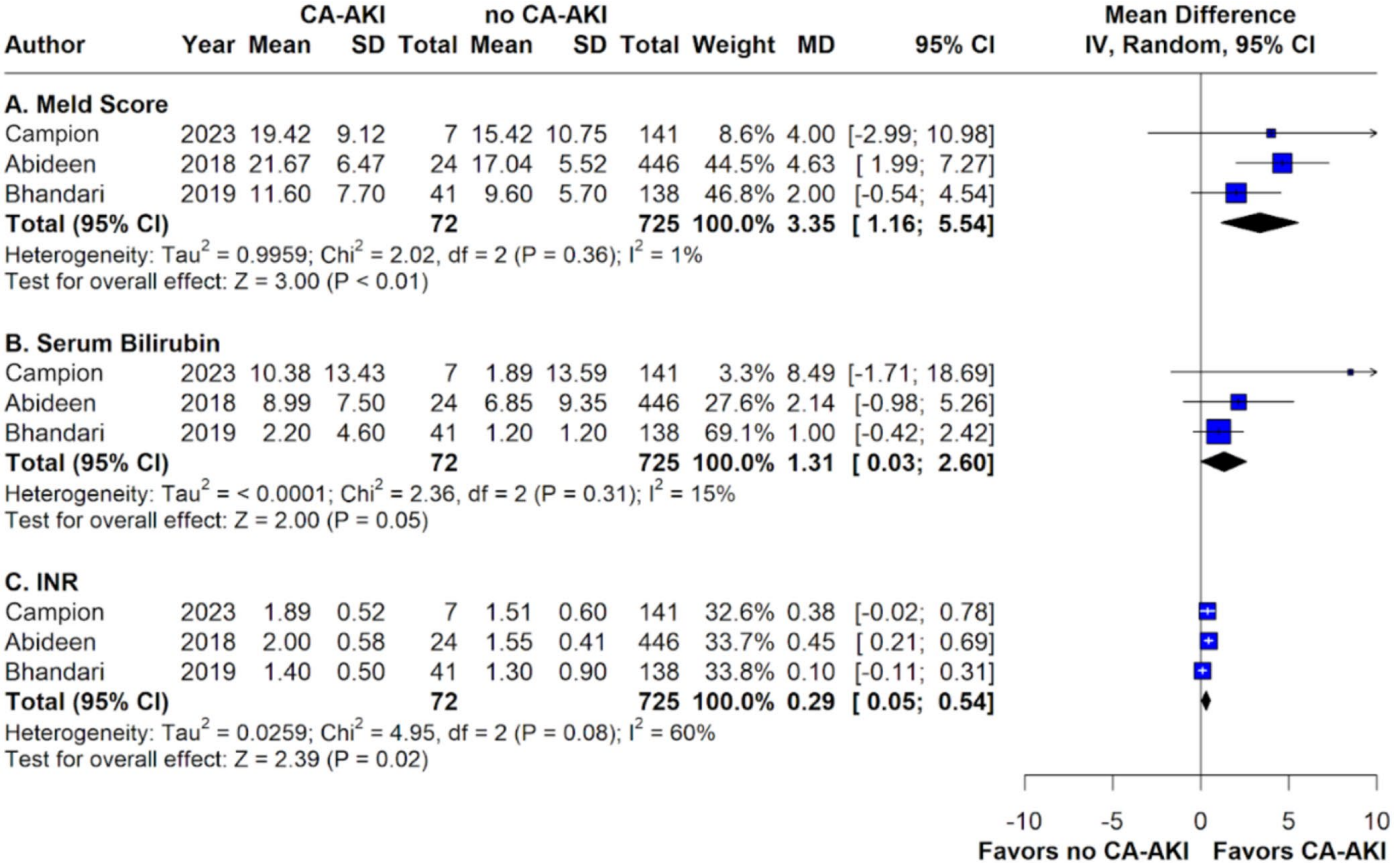
Comparison of baseline MELD score, serum bilirubin, and INR between iodinated contrast‐exposed patients with cirrhosis who developed CA‐AKI versus those who did not.

In the pooled analysis, patients who developed CA‐AKI exhibited significantly higher baseline values of MELD score (MD 3.4 points; 95% CI 1.2–5.5; *I* ^2^ = 1%; Figure 5A), serum bilirubin (MD 1.3 mg/dL; 95% CI 0.03–2.6; *I* ^2^ = 15%; Figure 5B), and INR (MD 0.3; 95% CI 0.1–0.5; *I* ^2^ = 60%; Figure 5C).

Male sex was not significantly associated with higher odds of developing CA‐AKI compared to female sex (OR 0.6; 95% CI 0.2–1.5; *I* ^2^ = 71%; Figure S1A). Similarly, there was no association between the presence of diabetes mellitus at baseline and the development of CA‐AKI (OR 0.9; 95% CI 0.6–1.4; *I* ^2^ = 13%; Figure S1B).

### 3.5 Sensitivity Analysis

The leave‐one‐out analysis did not identify any single study as a source of heterogeneity in the single‐arm forest plot of AKI prevalence among patients with cirrhosis exposed to contrast, or in the analysis of male sex as a risk factor. However, excluding the study by Bhandari et al. from the pooled analysis of INR outcomes eliminated the heterogeneity, while still confirming INR as a significant risk factor for CA‐AKI (MD 0.4; 95% CI 0.2–0.6; *I* ^2^ = 0; Figure S2).

### 3.6 Quality Assessment

Among the studies assessed using the ROBINS‐I tool, three were deemed to have a moderate risk of bias, while two were classified as having a serious risk of bias due to limitations in confounding adjustment and selection of reported results (Figure S3).

## 4 Discussion

Our study found a pooled prevalence of AKI of approximately 11% in patients with cirrhosis exposed to IC media, which is significantly higher than the 6.4% reported in a previous meta‐analysis that considered the general population [6]. Patients with cirrhosis are susceptible to AKI from multiple causes, including hypoperfusion, intrinsic kidney injury, and postrenal obstruction. AKI in patients with cirrhosis generally follows the development of portal hypertension through increased intrahepatic resistance and vasodilation of splanchnic vascular beds secondary to bacterial translocation and systemic inflammation [19]. Vasodilation leads to a decrease in effective central blood volume that, in turn, leads to activation of sodium/water conservation and vasoconstrictive neurohumoral pathways [20].

Hypoperfusion due to hypovolemia accounts for nearly half the cases of AKI in patients with cirrhosis, while intrinsic causes, primarily acute tubular necrosis, contribute to about 30% of cases [21]. Hepatorenal syndrome (HRS) accounts for approximately 15 to 20%, and less than 1% of cases are attributable to postrenal obstruction [7, 22]. When primary causes are excluded, contrast‐induced nephrotoxicity may be considered as a diagnosis of exclusion, attributed to the direct renal tubular toxicity of radiocontrast agents. Current consensus guidelines recommend that contrast agents should not be withheld when the diagnostic benefits outweigh the risk of AKI [7]. Our findings suggest that the elevated incidence of AKI in patients with cirrhosis deserves greater consideration in these decisions and may justify a more individualized, case‐by‐case approach.

Contrast media can induce kidney damage through multiple mechanisms. A predominant factor in the pathophysiology of CI‐AKI is the presence of contrast in the renal medulla, which disrupts the balance between oxygen supply and utilization due to reduced perfusion [23]. Contributing mechanisms for CI‐AKI include direct cytotoxic effects, obstructive injury to renal tubules, and damage from reactive oxygen species [23]. Prior studies involving the general population showed no difference in the incidence of AKI between groups, which leads us to think that patients with cirrhosis are particularly sensitive to contrast‐associated kidney damage [6].

In our study, the presence of ascites was the only factor associated with an increased risk of CA‐AKI in patients with cirrhosis exposed to contrast. In the context of cirrhosis and portal hypertension, ascites results from splanchnic and systemic vasodilation, leading to decreased circulating volume and renal hypoperfusion. The subsequent activation of the renin‐angiotensin‐aldosterone system (RAAS) promotes increased fluid retention [24]. This maladaptive hemodynamic response can precipitate HRS, a severe and rapidly progressive form of AKI that can occur in patients with decompensated cirrhosis and ascites [25]. When patients with HRS are exposed to contrast agents, the resulting renal medullary hypoxia may further aggravate kidney injury, leading to worse outcomes [26].

Excluding one notably heterogeneous study in the leave‐one‐out analysis significantly altered the results, showing that patients with cirrhosis receiving IC had a higher risk of AKI compared to those not receiving IC (OR 2.5; 95% CI 1.4–4.4). We propose that the observed heterogeneity in the study by Khan et al. may be explained by a higher proportion of inpatients in the control group (33.3%) compared to the IC group (26%). As noted in their study, inpatient status was independently associated with the development of AKI, which could help explain the higher AKI rates in the control group.

CKD has been a major concern for patients undergoing IC procedures, primarily due to the potential increased risk of CI‐AKI and CA‐AKI [27]. However, a recent meta‐analysis demonstrated that CKD does not significantly increase the risk of AKI in hospitalized patients with liver cirrhosis [28]. Similarly, our results indicate that CKD does not elevate the risk of AKI even when contrast is administered to these patients. This finding suggests that while CKD is generally a major risk factor for nephrotoxicity, its impact may not be as pronounced in patients with cirrhosis exposed to contrast, possibly due to more protective measures implemented in their clinical management. While diuretics are associated with pre‐renal AKI, our results also suggest that diuretic use alone does not significantly increase the risk of CA‐AKI in patients with cirrhosis.

As there is no specific treatment for CI‐AKI, many studies have been conducted on preventing this adverse effect [29]. Effective prophylactic measures for CI‐AKI include minimizing the volume of contrast administered and providing adequate intravenous fluid hydration to flush renal tubules and aid in the movement of the water‐soluble contrast material through the nephrons [30]. However, these studies mainly include the general population. The impact of prevention in patients with cirrhosis was not analyzed in our study due to insufficient data.

Our study has certain limitations. First, all the included articles were observational studies, which inherently carry limitations intrinsic to this design. Although randomized controlled trials would provide the highest level of evidence, their implementation in this setting is ethically challenging, and the contrast administration in patients with cirrhosis is typically driven by clinical necessity rather than research protocols. Therefore, the available evidence is largely derived from observational studies, which represent the most feasible study design for evaluating this question. Another limitation is the variability in AKI definitions across the included studies, reflecting changes in diagnostic criteria over time and potentially contributing to clinical heterogeneity in the pooled estimates. Notably, the included studies were also heterogeneous regarding cirrhosis severity, ranging from exclusively stable compensated patients to exclusively decompensated cohorts, as well as in their inclusion and reporting of concomitant active infections. As a result, significant between‐study heterogeneity was observed for some outcomes, although leave‐one‐out sensitivity analyses were conducted to identify the primary sources of variability. Finally, many studies did not report the type or dose of IC administered, limiting our ability to evaluate the influence of contrast exposure on AKI risk.

## 5 Conclusion

Our meta‐analysis showed that patients with cirrhosis receiving IC agents had a higher risk of AKI compared to those not receiving IC. Those who developed CA‐AKI had significantly more ascites and higher baseline MELD scores, driven by higher serum bilirubin and INR levels. These findings emphasize the need for caution and enhanced monitoring in this patient group. Additional studies with multivariate analysis and more rigorous inclusion criteria are needed to further strengthen the evidence.

## Funding

The authors have nothing to report.

## Ethics Statement

The authors have nothing to report.

## Consent

The authors have nothing to report.

## Conflicts of Interest

The authors declare no conflicts of interest.

## Supporting information


**Figure S1:** Effect of male sex and diabetes mellitus status on the risk of CA‐AKI in patients with cirrhosis following iodinated contrast exposure.
**Figure S2:** Leave‐one‐out sensitivity analysis of baseline characteristics in iodinated contrast‐exposed patients with cirrhosis who developed CA‐AKI versus those who did not.
**Figure S3:** Risk of bias assessment of included studies.

**Table S1:** Acute kidney injury (AKI) definitions of included studies.

## Data Availability

The data that support the findings of this study are available from the corresponding author upon reasonable request.
