# Supplementary material for: Acute Kidney Injury in Patients With Cirrhosis Exposed to Iodinated Contrast: A Systematic Review and Meta‐Analysis
Source: JGH Open. 2026 Sep 18;10(9):e70474. doi: 10.1002/jgh3.70474 (PMC13588755; doi:10.1002/jgh3.70474)
Supplement: Supplementary file 1 [file JGH3-10-e70474-s001.docx]

**Supplementary Table 1.** Acute kidney injury (AKI) definitions of included studies.

| **Study, Year** | **AKI definition** |
| --- | --- |
| Abideen, 2018 | “CI-AKI was defined according to the kidney disease improving global outcome (KDIGO) criteria as a rise in the serum creatinine by ≥ 0.3 mg/dL (≥26.5 µmol/L) within 48 hours.” |
| Bhandari, 2019 | “CIAKI was defined as a SCr increase of either ≥25% or ≥0.5 mg/dL from baseline within 72 hours, with the highest SCr within 72 hours being used to determine whether CIAKI occurred.” |
| Campion, 2023 | “AKI was defined as per KDIGO guidelines as modified in ICA-AKI criteria.” |
| Filomia, 2016 | “The International Club of Ascites has recently further stressed the association between AKI and cirrhosis, also adopting the definition by the AKI Network which defined AKI as an increase in serum creatinine (sCr) ≥0.3 mg/dL or ≥50% in 2 measurements 48 hours apart. [...] AKI network criteria were employed to assess contrast-induced AKI (CI-AKI) development.” |
| Khan, 2020 | “AKI was defined and classified using the Kidney Disease Improving Global Outcomes (KDIGO) classification. KDIGO classification defines AKI as either an increase in serum creatinine by ≥0.3 mg/dL within 48 hours, an increase in serum creatinine ≥1.5 times baseline which is known or presumed to have occurred within the prior 7 days, or decreased urine production to <0.5 mL/kg/hour for at least 6 hours.” |
| Lodhia, 2009 | “The aim of this study was to evaluate the development of CIN, which we defined as a decrease in CrCl of 25% or greater, temporally associated with the use of contrast.” |
| Najjar, 2002 | “RCIN is the most common cause of the hospital-acquired nephrotoxic acute renal failure, exceeding aminoglycoside-induced nephrotoxicity. It is defined as an acute deterioration in renal function in which Cr rises 24–72h after IVRC exposure. [...] Two of the cirrhotic patients (2.8%) and one patient in the control group (1.4%) developed RCIN. [...] Their clinical courses are shown in Figure 1.” |
| Safi, 2015 | “As summarized by the KDIGO guidelines on contrast-induced acute kidney injury13 the end-point was fulfilled if there was either an increase in creatinine by 44 mol/l or by 25% of baseline serum creatinine (SCr) or a drop in estimated glomerular filtration rate (eGFR) (as assessed by the MDRD 4 equation) by 25% of baseline14 within 72 h of contrast exposure.” |
| Tergast, 2022 | “Patients that received an emergency CM-CT were not considered for this study. Presence of AKI and severe AKI (Grade III AKI) were defined according to the criteria of the international ascites club.” |

AKI, acute kidney injury; CI, contrast-induced; CA, contrast-associated; CIN, contrast-induced nephropathy; CM-CT, contrast medium for computer tomography; Cr, creatinine; CrCl, creatinine clearance; eGFR, estimated glomerular filtration rate; ICA, International Club of Ascites; IVRC,

intravenous radiocontrast; KDIGO, Kidney Disease Improving Global Outcomes; MDRD, Modification of Diet in Renal Disease; RCIN, radiocontrast-induced nephropathy; SCr, serum creatinine.
