# Supplementary material for: Acute Kidney Injury in Patients With Cirrhosis Exposed to Iodinated Contrast: A Systematic Review and Meta‐Analysis
Source: JGH Open. 2026 Sep 18;10(9):e70474. doi: 10.1002/jgh3.70474 (PMC13588755; doi:10.1002/jgh3.70474)
Supplement: Supplementary file 2 [file JGH3-10-e70474-s002.docx]

# Supplementary Figure 1. Effect of male sex and diabetes mellitus status on the risk of CA-AKI in patients with cirrhosis following iodinated contrast exposure.

# Supplementary figure 2. Leave-one-out sensitivity analysis of baseline characteristics in iodinated contrast-exposed patients with cirrhosis who developed CA-AKI versus those who did not.

# Supplementary figure 3. Risk of bias assessment of included studies.
